# Is Vestibular Meniere's Disease Associated With Endolymphatic Hydrops?

**DOI:** 10.3389/fsurg.2020.601692

**Published:** 2020-12-18

**Authors:** Yuka Morita, Kuniyuki Takahashi, Shinsuke Ohshima, Chihiro Yagi, Meiko Kitazawa, Tatsuya Yamagishi, Shuji Izumi, Arata Horii

**Affiliations:** Department of Otolaryngology, Head and Neck Surgery, Graduate School of Medical and Dental Sciences, Niigata University, Niigata, Japan

**Keywords:** Meniere's disease, endolymphatic hydrops (EH), episodic vertigo, magnetic resonance imaging, diagnosis

## Abstract

**Background:** Vestibular Meniere's disease (American Academy of Ophthalmology and Otolaryngology, 1972) also known as possible Meniere's disease (American Academy of Otolaryngology Head and Neck Surgery, 1995) or vestibular type of atypical Meniere's disease (V-AMD) (Japan Society for Equilibrium Research, 2017) is characterized by an episodic vertigo without hearing loss. Though named as Meniere's disease (MD), this entity may not be caused solely by endolymphatic hydrops (EH).

**Objective:** To estimate the role of EH in vestibular Meniere's disease in comparison with definite Meniere's disease.

**Methods:** Thirty patients with unilateral definite MD and 16 patients with vestibular Meniere's disease were included. Those who met the criteria for definite or probable vestibular migraine were excluded. All patients underwent vestibular assessments including inner ear MRI 4 h after intravenous gadolinium injection, bithermal caloric testing, directional preponderance of vestibulo-ocular reflex in rotatory chair test, cervical- and ocular-vestibular evoked myogenic potential, stepping test, dizziness handicap inventory (DHI), and hospital anxiety and depression scale (HADS). All above tests and frequency/duration of vertigo spells were compared between vestibular Meniere's disease and MD.

**Results:** Even in unilateral MD, cochlear and vestibular endolymphatic hydrops (c-, v-EH) were demonstrated not only in the affected side but also in the healthy side in more than half of patients. Positive rate of v-EH in vestibular Meniere's disease (68.8%) was as high as that of MD (80%). In vestibular Meniere's disease, the number of bilateral EH was higher in the vestibule (56.3%) than that in the cochlea (25.0%). There were no differences in vestibular tests and DHI between vestibular Meniere's disease and MD; however, the frequency of vertigo spells was lower in vestibular Meniere's disease (*p* = 0.001). The total HADS score in the MD group was significantly higher than that in the vestibular Meniere's disease group.

**Conclusions:** MD is a systemic disease with bilateral involvement of inner ears. V-EH is a major pathophysiology of vestibular Meniere's disease, which would precede c-EH in the development of vestibular Meniere's disease, a milder subtype of MD. MRI is useful for differentiating MD from other vertigo attacks caused by different pathologies in bringing EH into evidence.

## Introduction

Meniere's disease is characterized by episodic vertigo, fluctuating progressive sensorineural hearing loss, and other cochlear symptoms such as tinnitus and aural fullness. The American Academy of Ophthalmology and Otolaryngology (AAOO) defined vestibular Meniere's disease as showing only episodic vertigo without hearing loss in 1972 (1). Paparella and Mancini reported two subgroups of vestibular Meniere's disease that had distinctive clinical profiles. In one subgroup, patients subsequently develop hearing loss and typical Meniere's disease with aural pressure, whereas those in the other subgroup do not (2). This suggests that subvarieties that include not only endolymphatic hydrops (EH) but also other pathophysiologies may be involved in vestibular Meniere's disease. Twenty-three years later, the term “vestibular Meniere's disease” was withdrawn from the American Academy of Otolaryngology-Head and Neck Surgery (AAO-HNS) definitions of Meniere's disease (3), probably because “vestibular Meniere's disease” had been thought to be not solely caused by endolymphatic hydrops. As a result, the formerly known vestibular Meniere's disease (1, 2) was placed into a category of “possible Meniere's disease” on the AAO-HNS 1995 criteria. Later definitions of Meniere's disease that were proposed by the Barany Society 2015 (4) and AAO-HNS 2000 (5) did not include the subvarieties of Meniere's disease, such as vestibular and cochlear Meniere's disease, whereas the definition by the Japan Society for Equilibrium Research (JSER) in 2017 included the vestibular type of atypical Meniere's disease (6), which is basically the same concept as that of vestibular Meniere's disease (1, 2).

Until 2007 when Nakashima et al. published the first report on visualization of endolymphatic hydrops in Meniere's patients (7), EH could only be evidenced by postmortem histology. To examine whether vestibular Meniere's disease is associated with EH, we conducted a comparative study to differentiate vestibular Meniere's disease from definite Meniere's disease by inner ear magnetic resonance imaging (MRI) studies as well as several assessments including demographic and clinical characteristics and vestibular function tests.

## Materials and Methods

The studies involving human participants were reviewed and approved by the Institutional Review Board of Niigata University Hospital (No. 2015-2440).

Thirty patients with unilateral definite Meniere's disease (Barany Society definition) (4) and 16 patients with vestibular Meniere's disease (AAOO-1972, Paparella and Mancini) (1, 2), diagnosed between 2015 and 2019, were included in this study. The side affected by definite Meniere's disease, that is, the side with a higher hearing threshold, was decided on the basis of an audiogram. In cases of vestibular Meniere's disease, we did not discuss the affected side but only concentrated whether the EH was identified unilaterally or bilaterally. All 16 patients with vestibular Meniere's disease did not meet the diagnostic criteria for both definite and probable vestibular migraine (VM) (8).

Demographic and clinical data, including age, sex, period from disease onset to consultation, frequency of vertigo spells, and duration of vertigo attack, were obtained from the patients' medical records.

**Figure 1:**
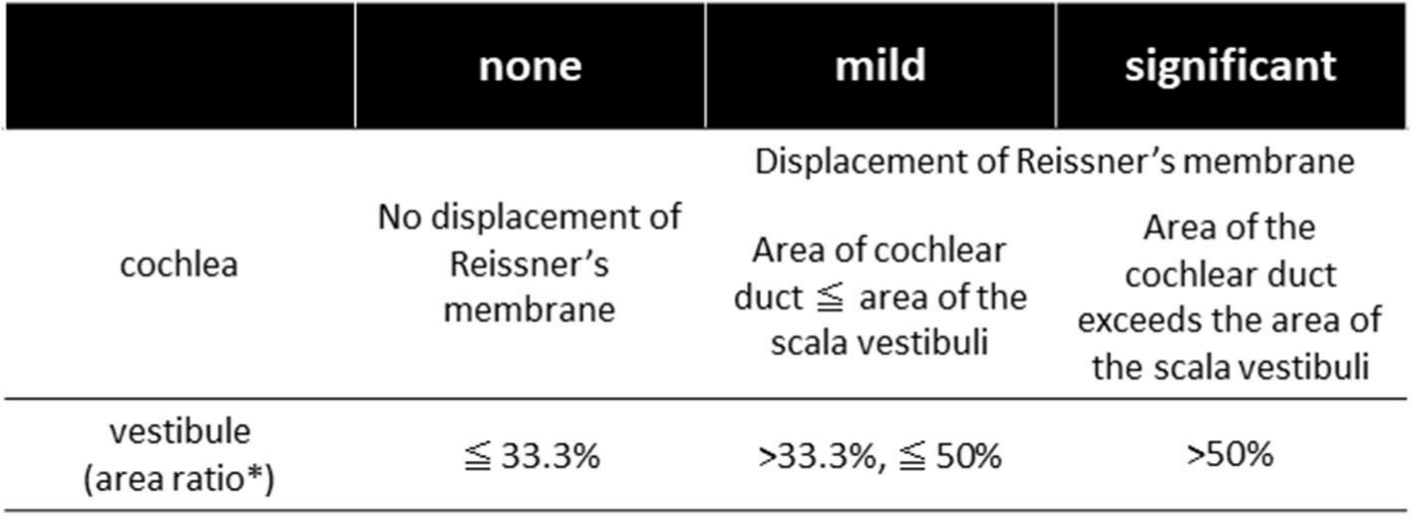
The grading system of endolymphatic hydrops [Nakashima et al. (10)]. In the vestibule, the grading was determined by the ratio of the area of endolymphatic space to the vestibular fluid space (sum of the endolymphatic and perilymphatic spaces). Patients with no hydrops have a ratio of one-third or less, those with mild hydrops have between one-third and a half, and those with significant hydrops have a ratio of more than 50%. In the cochlea, patients classified as having no hydrops show no displacement of Reissner's membrane; those with mild hydrops show displacement of Reissner's membrane, but the area of the endolymphatic space does not exceed the area of the scala vestibuli; and in those with significant hydrops, the area of the endolymphatic space exceeds the area of the scala vestibuli. *Ratio of the area of the endolymphatic space to that of the fluid space (sum of the endolymphatic and perilymphatic spaces) in the vestibule measured on image tracings.

All the patients underwent inner ear imaging studies using a 3-T MR unit after receiving intravenous contrast injections as described by Naganawa et al. (9). Briefly, MRI measurements were performed 4 h after intravenous administration of a single dose (0.2 ml/kg or 0.1 mmol/kg of body weight) of gadolinium-diethylenetriaminepentaacetic acid dimethylamide (Gadovist, Bayer Healthcare, Leverkusen, Germany) using a 3-T MR unit (MAGNETOM Prisma; Siemens, Erlangen, Germany) with a 64-channel array head coil. Heavily T2-weighted MR cisternography was obtained as the anatomical reference of the total lymph fluid, and heavily T2-weighted(hT2W)-3D-FLAIR with inversion time (TI) of 2,250 ms (PPI) and hT2W-3D-inversion recovery with TI of 2,050 ms (PEI) were obtained as the methods proposed by Naganawa et al. (9). Two head and neck radiologists with 30 and 22 years of experience who were blinded to the clinical data independently assessed the cochlear and vestibular endolymphatic hydropses (c-EH and v-EH) as none, mild, or significant, using a grading system proposed by Nakashima et al. (10) (Figure 1). Mild or significant hydrops was defined as positive hydrops. Discrepant interpretations were resolved by discussion between the radiologists.

The patients also underwent vestibular assessments, including bithermal caloric testing, cervical and ocular-vestibular-evoked myogenic potentials (VEMP), stepping test, and rotatory chair test. The validated Japanese versions of the Dizziness Handicap Inventory and Hospital Anxiety and Depression Scale were used for the evaluation of subjective symptoms and psychological state.

### Caloric Test

An alternate bithermal (26 and 45°C) air caloric test was performed. The maximum slow phase velocity of the nystagmus was measured on electronystagmography, and the canal paresis % (CP%) was calculated using the formula of Jongkees (11). A CP of >20% was considered to indicate a significant unilateral caloric weakness.

### VEMP

To quantify otolithic function, cervical VEMP (cVEMP) and ocular VEMP (oVEMP) were recorded using the Neuropack system (Nihon Koden, Japan). During the recording for cVEMP, the subjects were asked to lie in the supine position with their heads raised. The click (0.1-ms rarefactive square waves of 105-dB nHL) was used to induce cVEMP. For the recording of oVEMP, a hand-held electromechanical vibrator (Minishaker, Bruel & Kjaer, Denmark) fitted with a short bolt terminating in a plastic cap was used. The vibrator delivered a 500-Hz tone burst (4-ms plateau and 1-ms rise and fall) on the subject's skull at the Fz (midline of the hairline). The subjects were in the supine position during the measurement and asked to stare 30° upward and fix their gaze on a specific mark in front of them. The interaural asymmetry ratios (IAARs) of cVEMP and oVEMP were obtained using the following formula:

$$\begin{array}{l} \text{IAAR}=\left(\text{Ar}-\text{Al}\right)/\left(\text{Ar}+\text{Al}\right)\times \;100 \end{array}$$

Ar: normalized amplitude (p13-n23 or n10-p15) on the right side
Al: normalized amplitude (p13-n23 or n10-p15) on the left side

A |AR| of >33.3% was defined as a unilateral saccular (cVEMP) or utricular (oVEMP) dysfunction.

### Stepping Test

The subject was asked to stand upright with the feet close together in the centers of circles with radii of 0.5 and 1 m that were drawn on the floor. The subject was then blindfolded and instructed to stretch both arms straight forward and to step in place at a normal walking speed (~110 steps/min) for a total of 100 steps. The abnormality threshold was set as a rotational deviation >45°.

### Rotatory Chair Test

The rotatory chair test was performed using Nistamo21 IRN 2 (Morita, Japan). The patients sat in a rotatory chair to which a pendulum-like rotation was applied so that the maximum head angular velocity was 50°/s at a stimulation frequency of 0.1 Hz. The angular velocity of eye movements was monitored and analyzed. The vestibulo-ocular reflex directional preponderance (VOR-DP) was calculated, and a VOR-DP of >12% was considered a significant DP.

### Dizziness Handicap Inventory

The Dizziness Handicap Inventory (DHI) is a standard questionnaire that quantitatively evaluates the degree of handicap in the daily life of patients with vestibular disorders; it consists of 25 questions (12, 13). The total score ranges from 0 (no disability) to 100 (severe disability).

### Hospital Anxiety and Depression Scale

The Hospital Anxiety and Depression Scale (HADS) is a 14-item questionnaire that is comprised of two subscales for assessing non-somatic symptoms of anxiety (HADS-A) and depression (HADS-D). Each item of the questionnaire is rated from 0 to 3. The scores in the two subscales range from 0 (no sign of anxiety or depression) to 21 (maximum level of anxiety or depression). A score of ≥11 is indicative of probable anxiety or depression (14).

Statistical analyses were conducted using the SPSS version 21 package software. Described statistics are summarized in number, percentage, median and range, or mean and standard deviation. Intergroup comparisons were performed using the Mann–Whitney *U*-test. Intergroup comparisons of the qualitative variables were performed using the Fisher exact test. A threshold of *p* < 0.05 was set to evaluate statistical significance.

## Results

Table 1 shows the demographic and clinical characteristics and vestibular assessments of the patients with Meniere's disease and vestibular Meniere's disease. Vestibular Meniere's disease was female-dominant (*p* = 0.014) as compared with Meniere's disease. The period from the onset of the first vertigo attack to consultation in vestibular Meniere's disease (median: 72 months, range: 2–552 months) was significantly longer than that in Meniere's disease (median: 33 months, range: 2–408 months; *p* = 0.008). Vertigo spells usually occur once or twice a month in Meniere's disease but are significantly less frequent in vestibular Meniere's disease (*p* = 0.001). The total HADS score in the Meniere's disease group (15.2 ± 5.2) was significantly higher than that in the vestibular Meniere's disease group (10.1 ± 5.9). No significant differences in age, duration of vertigo attack, and DHI score were found.

**Table 1:** Demographic and clinical characteristics and vestibular assessments of the patients with Meniere's disease (MD) and vestibular Meniere's disease (VMD).

	**MD**	**VMD**	***p*-value**
Age	46 (19–83)	45 (25–71)	0.972
Male/female	15/15	2/14	0.014^*^
Period from onset, median (range)	33 (2–408)	72 (2–552)	0.008^*^
Duration of vertigo attack			0.329
<1 h	5	3	
<6 h	14	9	
<12 h	0	0	
>12 h	2	4	
Unknown	9	0	
Frequency of vertigo spells			0.001^**^
Every day	2	0	
1 or 2/week	1	0	
1 or 2/month	13	2	
5 or 6/year	5	2	
1 or 2/year	7	12	
Unknown	2	0	
DHI (mean ±*SD*)	41.1 ± 19.1	34.6 ± 15.0	0.321
P	10.8 ± 6.7	11.4 ± 3.8	0.624
E	14.5 ± 7.0	10.5 ± 7.3	0.059
F	15.7 ± 8.7	12.8 ± 7.2	0.186
HADS-total (mean ±*SD*)	15.2 ± 5.2	10.1 ± 5.9	0.008^**^
HADS-A	7.4 ± 3.4	5.1 ± 3.8	0.062
HADS-D	7.8 ± 3.3	5.1 ± 3.2	0.01^*^
**Vestibular Assessments**			
CP% > 20%	15 (50%)	4 (25%)	0.132
cVEMP asymmetry > 33.3%	9 (30%)	3 (18.8%)	0.323
oVEMP asymmetry > 33.3%	5 (16.7%)	1 (6.25%)	0.307
Stepping test positive	12 (40%)	9 (56.3%)	0.563
VOR-DP > 12%	12 (40%)	5 (31.3%)	0.502

* values indicates the statistical significant. *p < 0.05 and

** *p < 0.01*.

The vestibular function test revealed no significant differences in CP%, c-VEMPs, o-VEMPs, VOR-DP in the rotatory chair test, and stepping test score between the Meniere's disease and vestibular Meniere's disease groups.

**Figure 2:**
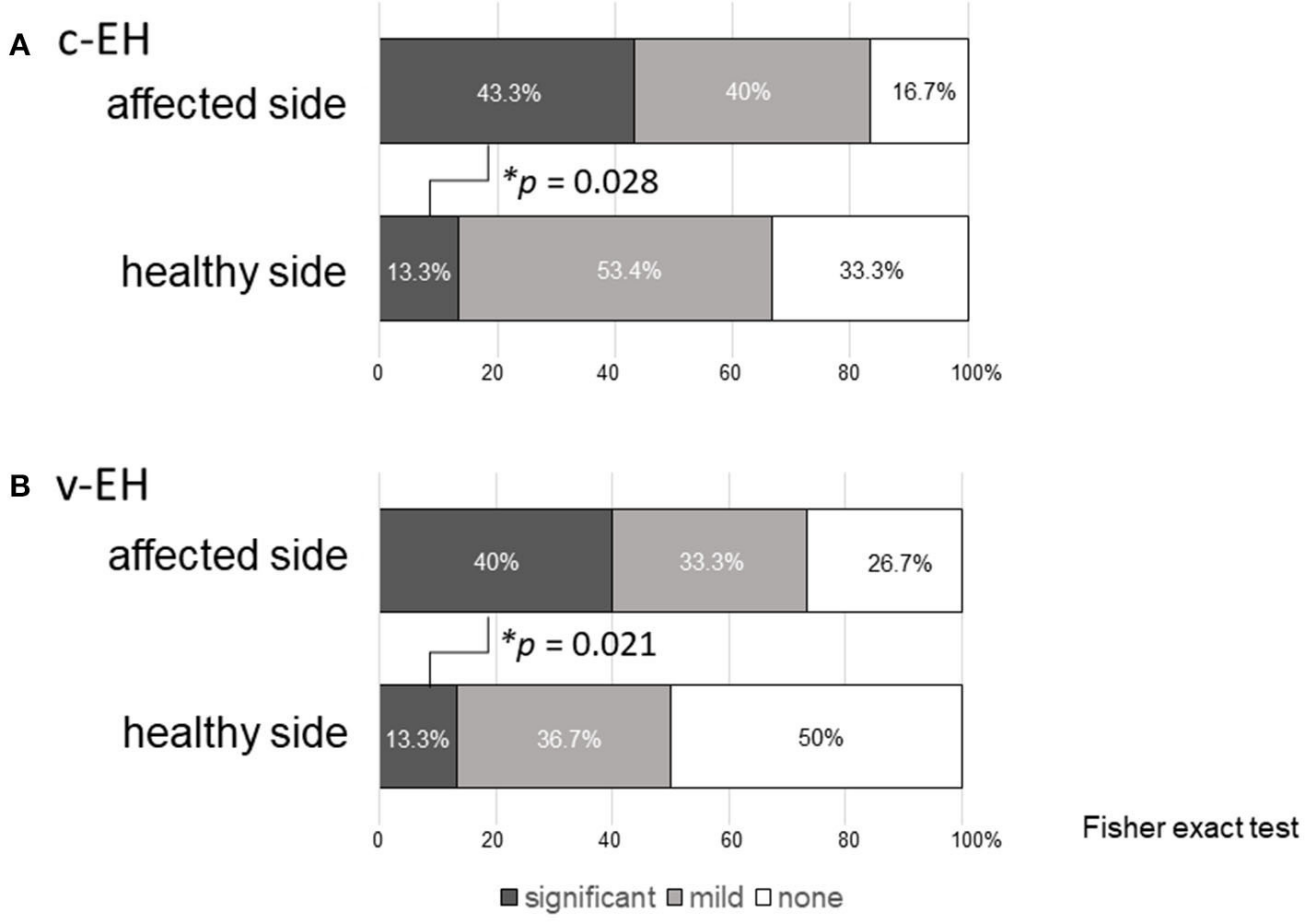
Endolymphatic hydrops in the affected and healthy side of Meniere's disease. **(A)** Cochlea endolymphatic hydrops (c-EH) and **(B)** vestibular endolymphatic hydrops (v-EH). Even on the healthy side, c-EH and v-EH (significant + mild) were demonstrated in 66.7% (13.3 + 53.4%; **A**, healthy side) and 50% (13.3 + 36.7%) of the patients in the Meniere's disease group (**B**, healthy side), respectively. The proportion of significant EH was higher on the affected side of the cochlea (**A**; *p* = 0.028) and vestibule (**B**; *p* = 0.021) than on the healthy side in the Meniere's disease group. c-EH, cochlea endolymphatic hydrops; v-EH, vestibular endolymphatic hydrops.

Figure 2 shows the statuses of (A) the cochlear EH (c-EH) and (**B**) vestibular EH (v-EH) of the affected and healthy sides of the patients in the Meniere's disease group. Even on the healthy side, c-EH and v-EH (significant + mild) were demonstrated in 66.7% (13.3 + 53.4%; Figure 2A, healthy side) and 50% (13.3 + 36.7%; Figure 2B, healthy side) of the patients in the Meniere's disease group, respectively. The proportion of significant EH was higher on the affected side of the cochlea (Figure 2A; *p* = 0.028) and vestibule (Figure 2B; *p* = 0.021) than on the healthy side in the Meniere's disease group.

**Figure 3:**
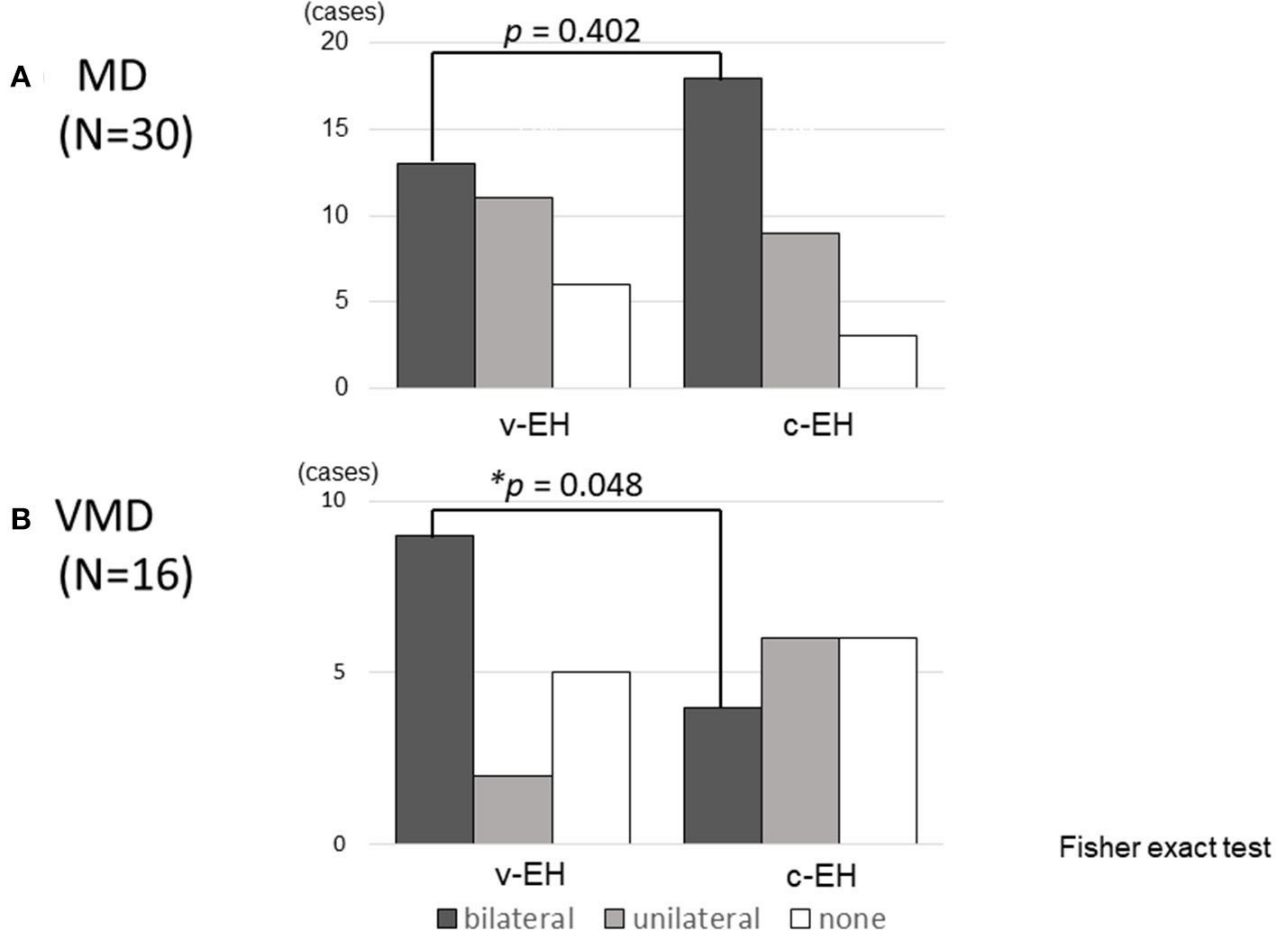
Cochlear and vestibular endolymphatic hydrops in **(A)** Meniere's disease (MD) and **(B)** vestibular Meniere's disease (VMD). **(A)** The number of bilateral v-EH was not different from bilateral c-EH in Meniere's disease. **(B)** The number of bilateral v-EH in v-EH-positive VMD patients was higher than that of bilateral c-EH in c-EH-positive VMD patients (*p* < 0.05). v-EH, vestibular endolymphatic hydrops; c-EH, cochlea endolymphatic hydrops.

Figure 3 shows the status of EH (bilateral, unilateral, or none) in the (A) Meniere's disease and (B) vestibular Meniere's disease groups. The number of bilateral v-EH cases in the v-EH-positive vestibular Meniere's disease group was higher than that of bilateral c-EH in the c-EH-positive vestibular Meniere's disease group (*p* < 0.05; Figure 3B). By contrast, the number proportion of bilateral v-EH was not different from that of bilateral c-EH in the Meniere's disease group (Figure 3A).

c-EH and/or v-EH was found in 93.3% of the patients with Meniere's disease and 87.5% of the patients with vestibular Meniere's disease (Table 2). While the positivity rate of c-EH in vestibular Meniere's disease (62.5%) was significantly lower than that in Meniere's disease (90%), the positivity rate of v-EH in vestibular Meniere's disease (68.8%) was as high as that in Meniere's disease (80%; Table 2).

**Table 2:** Endolymphatic hydrops in Meniere's disease (MD) and vestibular Meniere's disease (VMD).

	**MD**	**VMD**	***p*-value**
c-EH positive	27 (90%)	10 (62.5%)	0.034^*^
v-EH positive	24 (80%)	11 (68.8%)	0.308
c-EH and/or v-EH positive	28 (93.3%)	14 (87.5%)	0.221

c-EH, cochlear endolymphatic hydrops; v-EH, vestibular endolymphatic hydrops. * value indicate the statistical significant.

* *p < 0.05*.

## Discussion

### EH in Meniere's Disease

In general, Meniere's disease affects one side of the ears. Several studies reported that the incidence of bilateral Meniere's disease was 2–47%, depending on the length of follow-up and strictness of adherence to the criteria for diagnosis (15, 16). Recently, inner ear MRI scans have demonstrated the existence of EH in the unaffected ear in patients with Meniere's disease. Yoshida et al. reported that the incidence rates of EH in the cochlea and vestibule on the healthy side of patients with Meniere's disease were 46.9 and 53.2%, respectively (17). In our study, even on the healthy side, c-EH (Figure 2A, healthy side) and v-EH (Figure 2B, healthy side) were demonstrated in 66.7 and 50% of the patients with Meniere's disease, respectively. Taken together, these findings could imply that Meniere's disease is a systemic disorder or that an abnormal process affecting one ear can affect the other ear after time. As EH on the affected side was more severe than that on the healthy side (Figure 2A for c-EH and Figure 2B for v-EH), Meniere's disease may not have bilateral EH from the early stage but could progress from a unilateral disease to a bilateral disease.

### EH in Vestibular Meniere's Disease

As shown in Table 2, inner ear MRI studies demonstrated that v-EH was present in most patients (68.8%) with vestibular Meniere's disease. Kato et al. also reported that the positivity rate of v-EH in patients with vestibular Meniere's disease was 83% (18). Although healthy controls were not enrolled in this study, a previous report demonstrated that v-EH is rarely found (7%) in controls without audiovestibular diseases (17), suggesting that most patients with vestibular Meniere's disease are actually associated with v-EH. c-EH was also demonstrated in 62.5% of the patients with vestibular Meniere's disease, resulting in an EH-positivity rate of 87.5% in the vestibule and/or cochlea in patients with vestibular Meniere's disease. This rate was as high as that of definite Meniere's disease (93.3%; Table 2), which suggests that vestibular Meniere's disease is a vestibular type of primary hydropic ear disease (19). Attyé et al. also reported that half of the patients with repeated peripheral vertigo that lasted 20 min without hearing loss, which seems to have the same symptoms as vestibular Meniere's disease, showed v-EH and/or c-EH on MRI, which suggests an association of v-EH with vestibular Meniere's disease (20).

Not only v-EH but also c-EH was found in 62.5% of the patients with vestibular Meniere's disease (Table 2), which suggests that vestibular Meniere's disease could potentially develop to Meniere's disease. As shown in Figure 3B, the number of bilateral v-EH cases was higher than that of bilateral c-EH cases in patients with vestibular Meniere's disease, which suggests that v-EH precedes c-EH in the development of vestibular Meniere's disease. When c-EH starts to exert cochlear symptoms, vestibular Meniere's disease would develop into definite Meniere's disease.

### Differential Diagnosis of Vestibular Meniere's Disease

As a differential diagnosis of vestibular Meniere's disease, vestibular migraine (VM), which also shows episodic vertigo plus migraine, is most challenging to diagnose in clinical settings. Although we excluded patients with VM from the study by carefully interviewing patients regarding headache and migraine, patients with similar but not exactly the same pathophysiology as VM might be included in the study group. Our female-dominant study population (Table 1) would be partly consistent with the demographic characteristics of VM (21). This would account for the v-EH-negative populations of patients with vestibular Meniere's disease, which accounted for 31.2% of the patients with vestibular Meniere's disease (Table 2). According to a previous report that focused on the inner ear MRI of patients with VM, the positivity rate of EH in patients with VM was significantly lower than that in vestibular Meniere's disease (22). Taken together, the v-EH on inner ear MRI could be a useful sign for the discrimination of EH-associated vestibular Meniere's disease from VM.

Vestibular Meniere's disease might have two variants: one that subsequently develops into definite Meniere's disease and another that does not (2). The former group would be associated with EH, whereas the latter group may have a VM-related pathophysiology. Comparative studies to examine clinical courses such as future development into Meniere's disease or VM should be conducted in EH-positive and EH-negative episodic vertigo patients without hearing loss. Because treatment strategy is totally different between the two variants (i.e., the same treatment as the definite Meniere's disease for EH-positive variants and calcium blockers for the VM-related pathologies, inner ear MRI is quite important not only to diagnose vestibular Meniere's disease but also to select the appropriate treatment).

### Clinical Characteristics and Vestibular Function of Patients With Vestibular Meniere's Disease

In this study, we conducted a comparative study to differentiate vestibular Meniere's disease from definite Meniere's disease by assessing several factors, including not only EH imaging studies but also demographic and clinical characteristics and vestibular tests. Among these distinguishing features, a longer period from onset to consultation, lower frequency of vertigo spells, and lower total HADS score were found in the vestibular Meniere's disease group as compared with the definite Meniere's disease group (Table 1). These results suggest that the overall symptoms of vestibular Meniere's disease are mild as compared with those of definite Meniere's disease. In parallel with these symptom scales, the positivity rate of c-EH was lower in the vestibular Meniere's disease group (62.5%) than in the definite Meniere's disease group (90%; Table 2). All these findings suggest that vestibular Meniere's disease may be a milder subtype of Meniere's disease.

### Limitations and Conclusions

One limitation of the study was that no control participants were enrolled. For instance, the positivity rate of v-EH in vestibular Meniere's disease should have been compared with that in control patients. However, at present, contrast-enhanced MRI is difficult to perform in healthy volunteers. Instead, we performed a comparative study with definite Meniere's disease to show the similarity of inner ear status between vestibular and of definite Meniere's disease. Nonetheless, according to the previous reports using the same inner ear MRI methods as the present study, the positivity rate of EH in healthy groups was 38.1% for c-EH and 7.1% for v-EH (16). Therefore, the positivity rates of c-EH (62.5%) and v-EH (68.8%) in our patients with vestibular Meniere's disease seem sufficiently higher than those in healthy controls.

In conclusion, V-EH is a major pathophysiology of vestibular Meniere's disease, which would precede c-EH in the development of vestibular Meniere's disease, a milder subtype of Meniere's disease in terms of clinical symptoms and hydrops. Inner ear MRI is useful for the differential diagnosis of episodic vertigo without hearing loss.

## Data Availability Statement

The original contributions presented in the study are included in the article/supplementary materials, further inquiries can be directed to the corresponding author/s.

## Ethics Statement

The studies involving human participants were reviewed and approved by the institutional review board of Niigata University Hospital (No. 2015-2440). The patients/participants provided their written informed consent to participate in this study.

## Author Contributions

YM and AH contributed to the conception and design of the manuscript. KT, SO, CY, MK, TY, and SI made substantial contributions to the conception of the work and were responsible for data collection. YM wrote the manuscript. All authors contributed to manuscript revision, read, and approved the submitted version.

## Conflict of Interest

The authors declare that the research was conducted in the absence of any commercial or financial relationships that could be construed as a potential conflict of interest.
